# Photodynamic Inactivation of an Endodontic Bacteria Using Diode Laser and Indocyanine Green-Loaded Nanosphere

**DOI:** 10.3390/ijms22168384

**Published:** 2021-08-04

**Authors:** Naoya Higuchi, Jun-ichiro Hayashi, Masanori Fujita, Yuki Iwamura, Yasuyuki Sasaki, Ryoma Goto, Tasuku Ohno, Eisaku Nishida, Genta Yamamoto, Takeshi Kikuchi, Akio Mitani, Mitsuo Fukuda

**Affiliations:** 1 Department of Endodontics, School of Dentistry, Aichi Gakuin University, Nagoya 464-8651, Aichi, Japan; kinchan@dpc.agu.ac.jp (N.H.); wisteria@dpc.agu.ac.jp (M.F.); 2 Department of Periodontology, School of Dentistry, Aichi Gakuin University, Nagoya 464-8651, Aichi, Japan; yukiwa@dpc.agu.ac.jp (Y.I.); ysasaki@dpc.agu.ac.jp (Y.S.); gryoma@dpc.agu.ac.jp (R.G.); tasuku@dpc.agu.ac.jp (T.O.); enishida@dpc.agu.ac.jp (E.N.); genta@dpc.agu.ac.jp (G.Y.); tkikuchi@dpc.agu.ac.jp (T.K.); minita@dpc.agu.ac.jp (A.M.); fukuda-m@dpc.agu.ac.jp (M.F.)

**Keywords:** antimicrobial photodynamic therapy, indocyanine green, endodontics, *Enterococcus faecalis*

## Abstract

Apical periodontitis, an inflammatory lesion causing bone resorption around the apex of teeth, is treated by eradicating infectious bacteria from the root canal. However, it has a high recurrence rate and often requires retreatment. We investigated the bactericidal effect of antimicrobial photodynamic therapy (aPDT)/photodynamic antimicrobial chemotherapy (PACT) using indocyanine green (ICG)-loaded nanospheres coated with chitosan and a diode laser on a biofilm of *Enterococcus faecalis*, a pathogen of refractory apical periodontitis. Biofilm of *E. faecalis* was cultured in a porcine infected root canal model. ICG solution was injected into the root canal, which was then irradiated with a laser (810 nm wavelength) from outside the root canal. The bactericidal effect was evaluated by colony counts and scanning electron microscopy. The result of the colony counts showed a maximum 1.89 log reduction after irradiation at 2.1 W for 5 min. The temperature rise during aPDT/PACT was confirmed to be within a safe range. Furthermore, the light energy transmittance through the root was at a peak approximately 1 min after the start of irradiation, indicating that most of the ICG in the root canal was consumed. This study shows that aPDT/PACT can suppress *E. faecalis* in infected root canals with high efficiency.

## 1. Introduction

Antimicrobial photodynamic therapy (aPDT)/photodynamic antimicrobial chemotherapy (PACT) has been attracting attention as a promising method to eradicate pathogens from infectious lesions [1,2,3]. In theory with aPDT/PACT, reactive oxygen species (ROS), which are produced by irradiating light on a photosensitizer, demonstrate a bactericidal effect and suppress tissue infection. More specifically, the light-irradiated photosensitizer absorbs light energy and transitions to an excited singlet state in which the energy is enhanced. Because this state is unstable, most photosensitizers emit fluorescence and return to the ground state, but some transition to a triplet state because of the intersystem crossing of energy. When energy is transferred from a photosensitizer in a triplet state to a nearby triplet oxygen, the triplet oxygen is excited and becomes a singlet oxygen. This singlet oxygen destroys bacteria in infectious lesions [1,4,5]. In recent years, the drug resistance of bacteria caused by the overuse of antibiotics in the treatment of infectious diseases has become a serious problem. Therefore, the clinical application of aPDT/PACT, which can prevent the development of drug-resistant bacteria, has advanced as an alternative to antibacterial agents [6]. Other reported characteristics of aPDT/PACT include the suppression of endotoxin and protease activity, biological effects such as the anti-inflammatory benefits of the light itself, and the absence of side effects such as tissue damage.

Dentistry, along with dermatology, is a field in which aPDT/PACT is actively used [7]. A wide range of research has been conducted on periodontal treatment, treatment of peri-implantitis, endodontic treatment, treatment of tooth extraction sockets, and applications in oral cleaning [8,9,10,11]. aPDT/PACT has already been commercialized, and clinical applications continue to be developed in some countries. Methylene Blue (MB) [12,13] and Toluidine Blue O (TBO) [14,15] are currently used as photosensitizers, but there is still room for improvement in the extent of their bactericidal effect and their effect against biofilm, and basic research seeking more effective photosensitizers for dental applications is currently being conducted.

Endodontic disease and periodontal disease both cause destruction of periapical and periodontal tissue, including alveolar bone, and are major causes of tooth loss. Apical periodontitis, which is an endodontic disease that develops with the progress of dental caries, greatly affects the preservation of teeth. For the treatment of this disease, the infected root canal is typically disinfected as much as possible using sodium hypochlorite and calcium hydroxide. However, these medicaments have safety issues because they are strongly alkaline, and therefore an alternative method is needed. Additionally, the success rate of treatment even by specialists is reported to be only 70% or less because of the complexity of the root canal shape and the presence of bacteria that are difficult to eliminate and which may recur [16]. *Enterococcus faecalis* is strongly suspected to be related to refractory apical periodontitis, and this Gram-positive pathogen that remains in the root canal after endodontic treatment is thought to be one of the causes of recurrence [17].

Because sterilization of the root canal greatly affects the success or failure of infected root canal treatment, various medicaments have been studied. Sodium hypochlorite, which has been widely used in the past, is still an indispensable and effective medicament for chemical cleaning of root canals; however, safety problems such as serious medical accidents caused by misuse have been reported [18,19]. Calcium hydroxide, which is used as a disinfectant, is associated with misuse accidents [20], a low bactericidal effect for some bacterial species, and difficulty in use for root canal treatment of teeth requiring pulp regeneration treatment. Therefore, the development of a safe root canal cleaning method that does not use sodium hypochlorite or calcium hydroxide is important, and aPDT/PACT is considered to be a promising alternative treatment.

In endodontic aPDT/PACT, in addition to MB, TBO, Rose Bengal (RB), and curcumin (CUR), indocyanine green (ICG) has been used as a photosensitizer, and many studies have investigated the application of these photosensitizers to cleaning and disinfecting root canals for their bactericidal and antibiofilm effects. As for photosensitizers other than those, aPDT/PACT with porphyrin and chlorin has been reported to be effective against *E. faecalis* biofilm, suppressing the maturation, and phthalocyanine was also found to be as effective as conventional aPDT/PACT at a low concentration against *E. faecalis* biofilms [21,22,23]. ICG is a photosensitizer that is considered to be safe for use in humans, with relatively good results reported for its use in endodontic treatment [24]. Pourhajibagher et al. reported that ICG has high antimicrobial activity on biofilms compared with other photosensitizers [25]. Furthermore, some studies have reported that the dosage of ICG could be adjusted to further enhance the bactericidal activity for endodontic pathogens or other bacteria [26,27]. However, because the number of studies is still small and the experimental conditions of such studies including the concentration and dosage of the photosensitizer, the type of light source, and the irradiation method differ, the consensus on the effectiveness of ICG is still unclear.

In recent years, we have been developing an aPDT/PACT system using ICG and a diode laser to treat marginal periodontitis [28,29,30]. One feature of our method is the use of ICG-loaded nanospheres coated with chitosan (ICG-Nano/c) as the original dosage form of a photosensitizer. The theory behind this method is that the chitosan coating would positively charge the photosensitizers and improve their binding to negatively charged bacteria. In this study, we conducted basic research investigating the bactericidal effect of this aPDT/PACT system against *E. faecalis* biofilm, assuming future clinical applications. Other notable features of this study are the creation of an infected root canal model to reproduce the biofilm environment and irradiation of laser light from outside the root canal orifice to improve safety compared with conventional intra-root-canal irradiation. The effect on the biofilm, the temperature change of the root surface, and changes in the intensity of the laser light at the apex were evaluated.

## 2. Results

### 2.1. Bactericidal Effects aPDT/PACT with ICG-Nano/c on Planktonic E. faecalis

**Figure 1:**
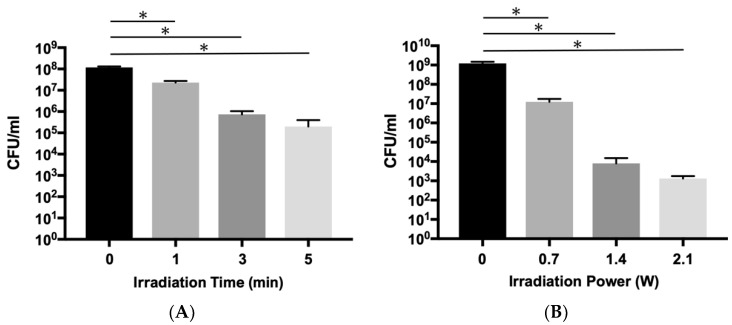
Bactericidal effect of aPDT/PACT using ICG-Nano/c on planktonic *E. faecalis*. (**A**) Bactericidal effect with irradiation of different durations (1, 3, and 5 min) indicated by viable colony counts. The diode laser was applied with a 0.7 W peak power output without air-cooling. (**B**) Comparison of the bactericidal effect with different irradiation powers (0.7, 1.4, and 2.1 W (0.49, 0.98 and 1.46 W/cm^2^)). The laser operated for 5 min without air-cooling. Data are presented as the mean ± standard deviation. Asterisks (*) denote *p* < 0.05 (*n* = 3).

First, the bactericidal effect of the aPDT/PACT system using ICG-Nano/c on planktonic *E. faecalis* was confirmed by colony counts. Because a bactericidal effect of 99% or more was observed for planktonic *P. gingivalis* with 1 min laser irradiation at a peak output power of 0.7 W (0.49 W/cm^2^) in previous studies using the current laser equipment, the same output was used to investigate the bactericidal effect on *E. faecalis*. When the irradiation time was 1 min, only approximately an 80.6% bactericidal effect was observed (0.71 log reduction), but 99.4% of the bacteria were killed in 3 min (2.21 log reduction), and 99.8% were killed in 5 min (2.76 log reduction) (Figure 1A and Table A1a). Next, the bactericidal effect was evaluated under varied irradiation outputs. When the output was doubled or tripled, a significant decrease in the number of detected bacteria was observed in a power-dependent manner (Figure 1B and Table A1b). The effects were 1.99 log reduction (98.9% killing) at 0.7 W, 5.18 (99.9993%) at 1.4 W (0.98 W/cm^2^), and 5.96 (99.99989%) at 2.1 W (1.46 W/cm^2^).

**Figure 2:**
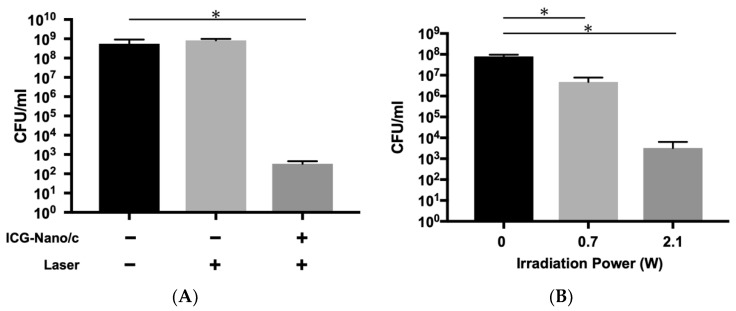
Confirmation that the bactericidal effect is due to aPDT/PACT. (**A**) Comparison of bactericidal effects with and without photosensitizers. The laser was applied at 2.1 W (1.46 W/cm^2^) for 5 min without air-cooling. A sufficient bactericidal effect could not be obtained in the absence of photosensitizers. (**B**) Bactericidal effect with air-cooling (2 L/min). A marked bactericidal effect was obtained, even when air-cooling reduced the effect of heat. Data are presented as mean ± standard deviation. Asterisks (*) denote *p* < 0.05 (*n* = 3).

To confirm that this bactericidal effect was due to aPDT/PACT, a comparison of the effect was made between groups with and without photosensitizers (Figure 2 and Table A2). While a significant decrease in the number of colony-forming units (CFUs) was observed in the aPDT/PACT group with photosensitizers, there was no bactericidal effect in the laser alone group without photosensitizers (Figure 2A). Additionally, we confirmed if the bactericidal effect was influenced by heat caused by the laser irradiation. As shown in Figure 2B, a sufficient bactericidal effect was observed when the laser was used with air-cooling to reduce the effects of heat. There was a 4.38 log reduction (99.996% killing) with irradiation at 2.1 W for 5 min.

### 2.2. Bactericidal Effects on E. faecalis Biofilm in the Infected Root Canal Model

**Figure 3:**
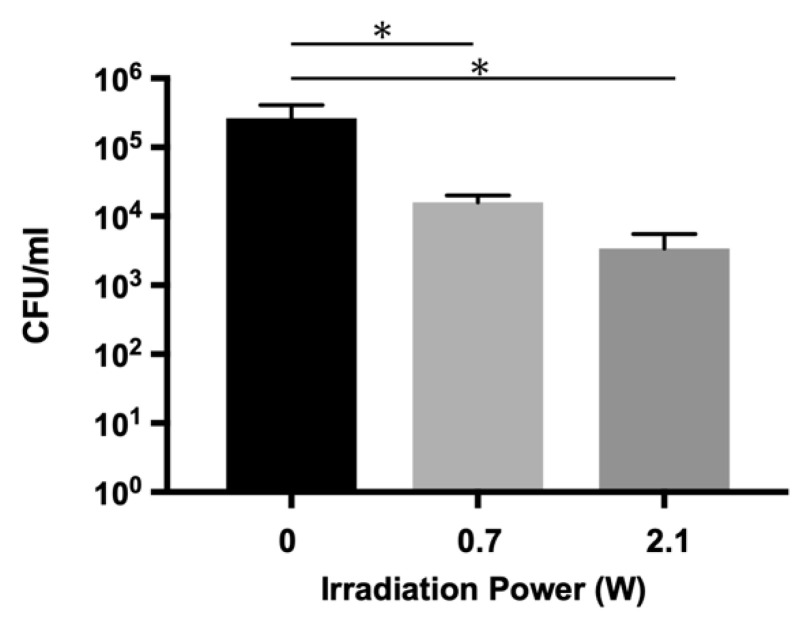
Bactericidal effect on *E. faecalis* biofilm in an infected root canal model. Lasers with different powers (0.7 W and 2.1 W (0.49 and 1.46 W/cm^2^)) were applied for 5 min with air-cooling. A marked bactericidal effect was observed at 2.1 W irradiation. Data are presented as the mean ± standard deviation. Asterisks (*) denote *p* < 0.05 (*n* = 3).

To verify the effect of aPDT/PACT in a biologically similar environment, an infected root canal model with an *E. faecalis* biofilm was prepared, and the sterilization experiment was performed. As shown in Figure 3 and Table A3, the number of bacteria detected in the root canals of the model was significantly lower in both aPDT/PACT groups than in the control group. A 1.89 log reduction (98.7%) was observed when the power of the irradiated laser was 2.1 W (1.46 W/cm^2^), while a 1.21 log reduction (93.9% killing) was obtained at 0.7 W (0.49 W/cm^2^) irradiation.

### 2.3. Scanning Electron Microscopy (SEM) Observations of E. faecalis Biofilm on Dentin Blocks after Treatment

**Figure 4:**
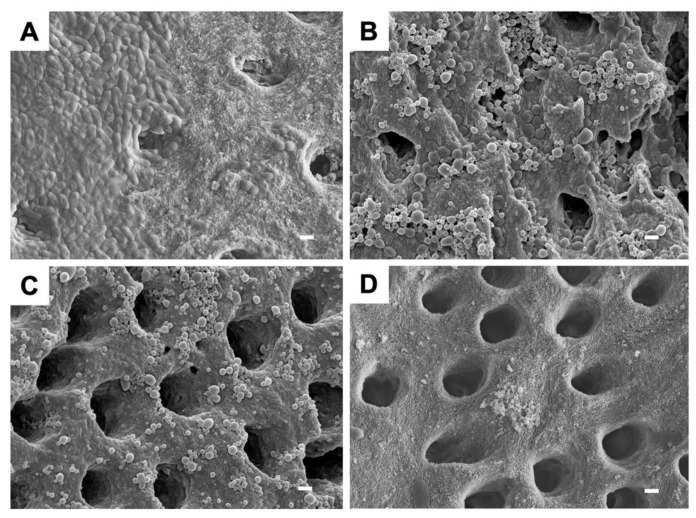
Scanning electron microscope images of *E. faecalis* biofilm after aPDT/PACT and NaOCl treatments. (**A**) Biofilm without irradiation is shown as a control. (**B**) Biofilm after aPDT/PACT with laser irradiation at 0.7 W. (**C**) Biofilm after aPDT/PACT with laser irradiation at 2.1 W (1.46 W/cm^2^). (**D**) Biofilm after treatment with NaOCl. All images are displayed at a magnification of ×5000. The length of the bar represents 1 µm.

An SEM image of the *E. faecalis* biofilm formed on a dentin block is shown in Figure 4A. The biofilm, which is composed of bacterial cells and extracellular polymeric substances, appeared to be spread in a thick sheet form covering the dentinal tubules. Figure 4B,C shows the images after aPDT/PACT. The biofilm appeared to be thinner and less abundant, and the dentinal tubules were exposed, but the morphology of the bacterial cells could still be confirmed. Furthermore, the nanoparticles of the photosensitizer were also observed to adhere to and aggregate with the remaining biofilm. The thinnest biofilm was observed in blocks treated with NaOCl, where the dentinal tubules could be clearly visualized in the blocks (Figure 4D).

### 2.4. Effect of Cooling on Temperature Elevation of the Root Surface during Laser Irradiation

**Figure 5:**
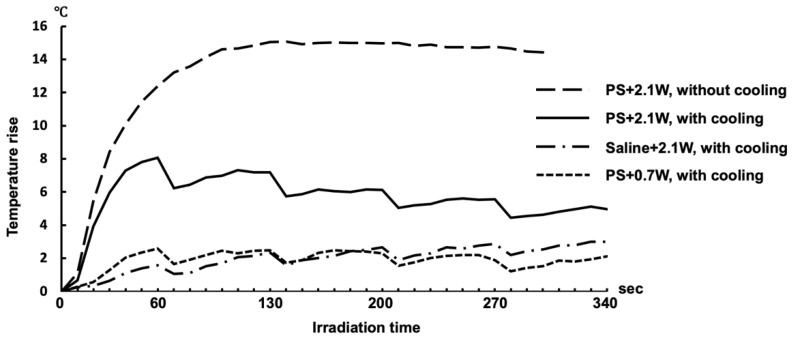
Temperature transitions on the root surface of extracted human teeth during laser irradiation. In the group without air-cooling, the laser was applied continuously for 5 min. In the air-cooled groups, the laser operated intermittently for 5 min with a 10 s rest period every minute. The average of the highest temperatures within a heat distribution captured by thermography is indicated in the polygonal line graph (*n* = 5).

For future clinical application of this aPDT/PACT system to human root canals, it is necessary to avoid damage to the tissues around the roots caused by heat generated by the laser irradiation. The temperature elevation of the root surface of extracted teeth was investigated with air-cooling and intermittent irradiation (Figure 5). When aPDT/PACT using a photosensitizer was performed with an output power of 2.1 W (1.46 W/cm^2^) without cooling, the temperature of the root surface increased by up to 15 °C. However, with cooling of the root surface at the same output, the maximum elevation in temperature was 8 °C. The maximum elevation in temperature was reached 60 s after the start of irradiation and then gradually decreased. However, when the root canal was filled with physiological saline without a photosensitizer, the temperature rose only 2.5 °C even when irradiated at 2.1 W. The temperature rose to the same level as when saline was used at 0.7 W.

### 2.5. Measurement of the Light Energy Transmitted through the Tooth Root

In aPDT/PACT, a singlet oxygen is generated by adding light energy to the photosensitizer pigment. However, when the photosensitizer is exposed to light, the pigment is thought to gradually decompose, and its function as a photosensitizer is diminished. In the aPDT/PACT method used in this study, a photosensitizer was injected into the root canal, and then light was irradiated from outside the orifice of the root canal. The light is mostly absorbed by the photosensitizer in the root canal and the dentin of the root canal wall but partly passes through the root. As the pigment is decomposed by light, the light energy absorbed by the photosensitizer decreases, and as a result, energy transmitted through the tooth root increases.

**Figure 6:**
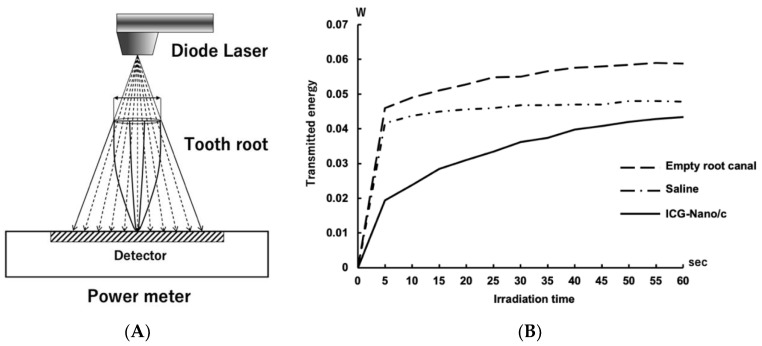
Transition of the light energy transmitted through the tooth root. (**A**) Schematic diagram of laser irradiation and detection. The guided circle of the irradiation range was matched to the outer circumference of the root. (**B**) The transition of the energy transmitted through the root is shown in a polygonal line graph. The value displayed in the power meter was recorded every 5 s (*n* = 5).

**Figure A1:**
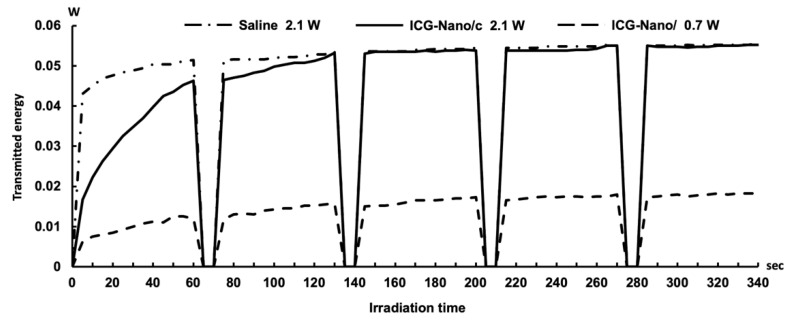
Transition of transmitted energy over the total irradiation time. The laser was irradiated on the tooth root intermittently for 5 min with a rest period of 10 s every minute. Canals of the root were filled with ICG-Nano/c, saline or none. The light energy transmitted through the tooth root was measured during full time irradiation and recorded every 5 s.

Therefore, the change in energy transmitted through the tooth root was measured during irradiation for 5 min to investigate whether the pigment decomposed (Figure 6A). Less energy was transmitted through the roots when the root canal was filled with saline than when the root canal was empty. At 5 s after irradiation, the level of energy that was transmitted through the root filled with ICG-Nano/c was approximately half of that transmitted through the root filled with saline. The level of energy that was transmitted gradually increased and was approximately equal to that of the control at 60 s (Figure 6B). Measurements were taken up to 340 s (a total of 5 min irradiation and 40 s rest), and the amount of transmitted energy was almost flat after 140 s (Figure A1).

## 3. Discussion

In this study, we investigated the bactericidal effect of aPDT/PACT using ICG-Nano/c on *E. faecalis*, a widely known pathogen of endodontic lesions such as refractory apical periodontitis. *E. faecalis* is also a pathogen of urinary tract infections [31], and its broad spectrum of resistance to antibiotics such as vancomycin is regarded as a public health problem [32]. Genomic analysis of vancomycin-resistant strains revealed that *E. faecalis* develops drug resistance by horizontal gene transfer mechanisms including transposons [33]. Therefore, sterilization methods that do not rely on antibiotics are required for the extermination of this bacteria. Our aPDT/PACT method showed a sufficient bactericidal effect on *E. faecalis* without dependence on antibacterial agents, which is of clinical significance.

The bactericidal effect on planktonic bacteria was investigated in relation to the irradiation energy and irradiation time. Our previous studies on a pathogen of marginal periodontitis confirmed that the aPDT/PACT method has a bactericidal effect of a 2 log reduction (1 min) to a 5 log reduction (5 min) against planktonic cells of *Porphyromonas gingivalis* [28]. However, when testing the bactericidal effects on planktonic cells of *E. faecalis* with the same output (0.7 W, duty cycle of 50%, 0.49 W/cm^2^) in this study, the effect was less than a 1 log reduction at 1 min and approximately a 2 log reduction even at 5 min; this was considered to be weaker than the effect on *P. gingivalis*. Therefore, when the energy output of the laser was increased to three times the original setting (2.1 W, 1.46 W/cm^2^), the same level of bactericidal effect as on *P. gingivalis* could be obtained. The reason why higher energy was required to exert a sufficient bactericidal effect on *E. faecalis* is considered to be related to the difference in the antioxidative mechanisms of both bacteria. *E. faecalis* is a Gram-positive facultative anaerobic bacterium, which is more resistant to oxygen than Gram-negative obligate anaerobic bacteria such as *P. gingivalis*, and numerous antioxidative enzymes have been identified in *E. faecalis* [34]. *E. faecalis* possesses manganese-containing superoxide dismutase, which is a classical ROS scavenging enzyme [35], and catalase of *E. faecalis* converts hydrogen peroxide to water and oxygen in a heme-dependent manner [36]. Additionally, *E. faecalis* is known to produce extracellular free radicals and exert pathogenicity [37]. Thus, because *E. faecalis* has various functions to resist oxidative stress, a higher output than that for *P. gingivalis* may have been required to achieve a sufficient bactericidal effect.

Many previous studies have focused on the bactericidal effect of aPDT/PACT against monospecies biofilms of *E. faecalis,* with variable results. Zand et al. reported that no living bacteria were detected in the biofilm-formed dentin block after aPDT/PACT treatment with TB [38]. Kishen et al. showed a bactericidal effect of a 5 log reduction by aPDT/PACT using MB and RB [39], and Akbari et al. showed a 2.81 log reduction with nanographene oxide including ICG [27]. However, a number of studies have reported that the bactericidal effect of aPDT/PACT on *E. faecalis* biofilm was less than 90%. According to Oda et al., aPDT/PACT with MB and CUR resulted in detection rates of living bacteria of 29.80% and 26.20%, respectively [40]. Golmohamadpour et al. applied three types of metal–organic frameworks including ICG for aPDT/PACT, resulting in detection rates of living bacteria from 45.12% to 62.67% [41]. Furthermore, potassium iodide-potentiated PDT with MB and RB increased bactericidal effects to 86.50% and 91.50%, respectively, in a study by Li et al. [42]. In this study, we used aPDT/PACT with ICG-Nano/c in an infected root canal model consisting of an *E. faecalis* monospecies biofilm in root canals. We detected a significant decrease in bacterial count, with a bactericidal effect of 98.7% (1.89 log reduction) (Figure 3). Because of differences in methodologies such as laser conditions, analysis methods, and biofilm production, a simple comparison of studies cannot be easily made, but it is apparent that aPDT/PACT with ICG-Nano/c produces a significant bactericidal effect. However, the bactericidal effect in our study did not exceed 99%. It was previously reported that mature (weeks old) biofilms experience a significantly lower bactericidal effect compared with immature (days old) biofilms [43]. Because we used an infected root canal model that involves a 3-week-old biofilm, the maturation of the biofilm may have impacted the extent of the bactericidal effect of aPDT/PACT.

Although quantitative measurement was not performed, we were able to observe an apparent biofilm reduction on SEM images (Figure 4). Extracellular polymeric substances form at least 90% of the total biofilm mass and enhance the structural complexity and strength of the biofilm [44]. Hence, extracellular polymeric substances are among the first lines of defense against antibiotic and biocide diffusion and thus reduce photosensitizer penetration. Various components of extracellular polymeric substances are subject to attack by ROS [45], which form from aPDT/PACT. The effect of ROS damage to the *E. faecalis* biofilm is evidenced by SEM imaging [46]. Chitosan can also damage the extracellular polymeric substances and disrupt the structure of biofilm [47]. Thus, it can be hypothesized that the chitosan in ICG-Nano/c and the ROS resulting from irradiation cause damage to the extracellular polymeric substances in biofilm.

The laser output used in aPDT/PACT on the *E. faecalis* biofilm was 2.1 W (duty cycle of 50%, 1.46 W/cm^2^), which was higher than that of a commercially available general aPDT/PACT system. It is known that ICG generates heat when excited by laser light [48]. Additionally, because laser light barely passes through the root dentin, the energy absorbed by dentin is converted into heat at a high rate. Therefore, the irradiated site was air-cooled to avoid heat injury, in consideration of future clinical applications. Previous research focusing on the temperature increase of the root canal surface caused by the vertical condensation root canal filling technique [49,50] and on the effect of elevated bone temperatures on living organisms [51] has established that a 10 °C increase in the root surface temperature may damage the periodontal tissues, cementum, periodontal ligament, and alveolar bone. In this study, we found that the temperature of the root surface reached a maximum at 60 s after irradiation in aPDT/PACT, but even the maximum output of 2.1 W averaged a temperature increase of only 8 °C. Additionally, after the maximum temperature elevation was observed, the temperature dropped immediately as a result of the 10-s resting time and air-cooling. Furthermore, the temperature of the root surface gradually decreased. These results are within the critical temperature elevation range of 10 °C. Therefore, little damage would have been sustained by the periodontal tissue given that the maximum temperature range lasted only 10–20 s.

When the empty root canal was irradiated with a laser, 0.06 W of energy was transmitted through the root (Figure 6). Because the irradiation energy was set to a peak output of 2.1 W and a duty cycle of 50%, the theoretical average energy irradiated on the root was 1.05 W. Therefore, approximately 94% of the energy was absorbed by the root dentin. In the root canals filled with ICG-Nano/c solution, the transmitted energy of the laser was 0.02 W at 5 s after the start of irradiation but gradually increased thereafter to 0.04 W at 1 min. This was almost the same value as when physiological saline was placed in the root canal. This result indicates that the ICG-Nano/c absorbed laser energy immediately after the start of irradiation, but the absorbance decreased over time. ICG decomposes in light [52]. The polymethine chain of ICG exposed to light forms a dioxane ring producing singlet oxygen, which is further decomposed into a carbonylated molecule. Therefore, the decrease in energy absorption is thought to be caused by the decomposition of ICG-Nano/c in the laser light, and its function as a photosensitizer is gradually lost. If most of the photosensitizer in the root canal is consumed within 1 min of the start of irradiation, 5 min of irradiation is longer than necessary. The reason why the bactericidal effect increased in a time-dependent manner against the planktonic *E. faecalis* in Figure 1 may be that a sufficient amount of photosensitizer was present around the bacteria. To establish a clinical application of this method in the future, it will be necessary to investigate the bactericidal effect under changing conditions, such as a smaller interval for the irradiation time and multiple injections of photosensitizer.

Cleaning the root canal with hypochlorous acid produces high bactericidal activity, and this medicament is still widely used today. In this study, hypochlorous acid showed a high biofilm removal effect (Figure 4D). However, the outflow from the apical foramen is regarded as a problem, and there is a demand for a safer method. Furthermore, conventional aPDT/PACT for bacterial decontamination of root canals recommends inserting the tip of the optical probe into the root canal, which raises safety concerns. The advantage of the method used in this study is that a high bactericidal effect equal to that of previous studies can be obtained without inserting a probe into the root canal. Irradiation was performed from 1 cm above the root canal orifice. It can be concluded that this method of aPDT/PACT functions well without inserting the laser tip into the root canal, as evidenced by the consumption of most of the photosensitizer injected into the root canal. Our findings suggest that this method is safer than conventional methods.

One limitation of this study is that it only validates the bactericidal effect against a single bacterial biofilm. Because biofilm usually consists of a complex bacterial population, it is possible that the reaction in actual root canals may not be the same as in the case of a single bacterial biofilm. Additionally, the effect of this method on apical periodontitis is unclear because no clinical evaluation was conducted. In fact, as can be seen in Figure 4D, our method shows less bactericidal effect than NaOCl. However, there is still room for improvement in this method, such as multiple injections of photosensitizer, and many problems remain to be solved to achieve clinical application. In our future research, we plan to optimize the method and evaluate its effect in clinical studies.

## 4. Materials and Methods

### 4.1. Preparation of ICG-Nano/c

ICG-Nano/c was Prepared by the Emulsion Solvent diffusion method in oil [53], following a protocol from Nagahara et al. [28].

### 4.2. Bacterial Strain

*Enterococcus faecalis* was used as the test organism because it is often found in persistent and retreatment cases [54]. The *E. faecalis* ATCC 19,433 strain was grown aerobically on brain–heart infusion (BHI) agar plates (Bacto; DIFCO Laboratories, Franklin Lakes, NJ, USA) at 37 °C. Single colonies were then inoculated into BHI broth and cultured aerobically to the mid-log phase at 37 °C. The number of bacteria was adjusted with sterilized saline spectrophotometrically to a cell density of approximately 10^8^ CFU per mL (optical density of 0.1 at 600 nm) before subsequent experiments. To summarize, 5 mg of ICG (Ophthagreen; Santen Pharmaceutical, Osaka, Japan), 100 mg of poly (lactic-co-glycolic acid), and 100 mg of Span80 (Kanto Chemical Co., Inc., Tokyo, Japan) solution, which was dissolved in 3 mL acetone and 1 mL of methanol, was added at 2 mL/minute to 100 mL of 2% hexaglycerin-condensed ricinoleate containing triglyceride and n-hexane. The total mixture was stirred at 35 °C for 3 h under a vacuum (400 rpm). It was then centrifuged to remove oil and n-hexane. After centrifugation, nanospheres were mixed with 50 mL of a solution of 2% poly-vinyl alcohol/0.5% chitosan to form a chitosan coating. The mixture was further centrifuged at 26,960× *g* for 10 min at 4 °C. The nanosphere cluster was suspended in mannitol solution to separate each particle and frozen at −45 °C for 2 h. Preparation of ICG-Nano/c was completed by freeze-drying for 48 h. Properties of the produced ICG-Nano/c were (1) it contained 5 mg/g ICG, (2) the average nanosphere size was 560 nm. ICG-Nano/c solutions were freshly made in sterilized saline at 20 mg/mL and used at a final concentration of 10 mg/mL.

### 4.3. Laser Application

LIGHTSURGE SQUARE (Osada, Tokyo, Japan) was used as a diode laser with a central wavelength of 810 ± 20 nm that can output up to 3 W. Other features of this laser are: (1) light is distributed through the fiber-optic applicator, (2) the diameter of the fiber core is 600 μm, and (3) the spread angle of emitted light is 20.49°. In this study, we set it to a repeated pulse mode with a pulse width of 100 msec, 50% duty cycle, and used it under various conditions (peak power outputs: 0.7–2.1 W, 0.49–1.46 W/cm^2^, irradiation time: 1, 3, 5 min). The light probe was placed 10 mm above the surface of the samples. The irradiation spot area was set to a diameter of 0.956 mm.

### 4.4. Preparation of the Infected Root Canal Model

**Figure 7:**
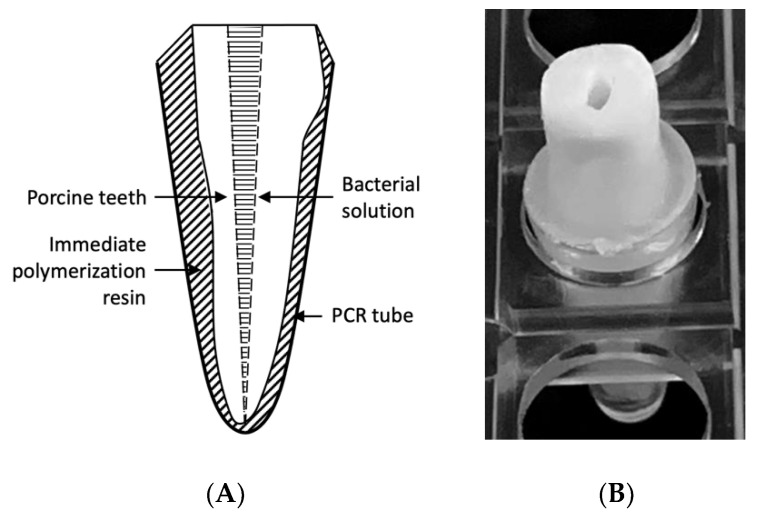
Preparation of the infected root canal model based on porcine teeth. (**A**) Schematic diagram of the infected root canal model. (**B**) Photograph of the infected root canal model. The root was separated from the first or second molar, and the length was adjusted to 13 mm. The root canal was cleaned mechanically and chemically, and then the root was autoclaved before it was fixed in a PCR tube with immediate polymerization resin. A bacterial solution of *E. faecalis* was inoculated in the root canal and incubated for 21 days at 37 °C.

An infected root canal model was prepared using extracted porcine teeth (Figure 7). Specifically, the roots of the first and second molars were separated and extracted, and the root length was adjusted to 13 mm. The root canals were then cleaned and shaped up to #60 with a K file (MANI, Inc., Utsunomiya, Japan) using conventional methods, chemically cleaned with sodium hypochlorite solution and EDTA solution, and sterilized in an autoclave. The apical foramen was closed with an immediate polymerization resin (Ortho Crystal; JM Ortho, Tokyo, Japan) and fixed in a polymerase chain reaction tube. The *E. faecalis* bacterial solution was inoculated into the root canal and cultured at 37 °C for 21 days. During the culture period, the medium was changed three times a week.

### 4.5. Bactericidal Assay on Planktonic Cells

The experimental group was treated with aPDT/PACT as follows: 100 µL of the bacterial solution and 100 µL of the ICG-Nano/c solution prepared to 20 mg/mL with sterile saline (ICG-Nano/c final concentration: 10 mg/mL) were added to a microtube and irradiated with the laser at intensities of 0.7, 1.4, and 2.1 W for 1, 3, and 5 min. After serial dilution, the total solution was spread on BHI agar plates, and a colony count was performed. There were three samples per condition. Two control groups were set up as follows: (1) a positive control group in which the same amount of sterile saline was added instead of the ICG-Nano/c solution and no laser irradiation was performed and (2) a laser alone group in which the same amount of sterile saline was added instead of the ICG-Nano/c solution and laser irradiation was performed.

### 4.6. Bactericidal Assay on the Biofilm in the Infected Root Canal Model

Using the completed infected root canal model, the components of the medium were washed away with 2 mL of sterile saline using a syringe (Terumo Syringe 2.5 mL SS-02SZ; Terumo, Tokyo, Japan) and an irrigation needle (Nishika Rootclin Needle 23G; Nishika, Yamaguchi, Japan). The root canals were then dried with paper points (#60) (Morita Paper Point; J. MORITA CORP., Suita, Japan). ICG-Nano/c solution was added to the experimental group (final concentration 10 mg/mL), and laser irradiation was performed while blowing air (2 L/min) from a nozzle built into the tip of the light probe. The irradiation intensities were 0.7 and 2.1 W. The irradiation time was 5 min, and irradiation was stopped for 10 s every minute. The two control groups were set up in the same way as the assay for planktonic cells. Paper points (#55) were placed for 1 min in the root canals. After the paper points were removed, serial dilution and colony counting were performed. There were three samples per condition.

### 4.7. SEM Observations of Biofilm on Dentin Blocks after aPDT/PACT Treatment

A dentin block (5 mm × 5 mm × 2 mm) was prepared from extracted human teeth. The block was chemically cleaned with sodium hypochlorite solution and EDTA solution, sterilized in an autoclave, immersed in the *E. faecalis* bacterial solution, and cultured aerobically at 37 °C for 21 days. After the block was washed with 5 mL of sterile saline using a syringe, aPDT/PACT treatment was performed on the experimental group. Irradiation was performed under the same conditions described in Section 4.6. Control groups were also set up with the same conditions described in Section 4.6. As NaOCl treatment, dentin blocks were placed in 3% NaOCl solution (Dental Antiformin; Nishika, Yamaguchi, Japan) for 1 min.

The samples were washed with 5 mL of sterile saline and fixed with a mixture of 4% paraformaldehyde and 5% glutaraldehyde for 24 h at 4 °C. The samples were washed again in the same manner, dehydrated with an ascending ethanol series (50, 60, 70, 80, 90, 95, 100, 100%), replaced with t-butyl alcohol, and freeze-dried for 4 days using a freeze-dryer (EYE4 FDS-1000 type; Tokyo Rika Kikai Co. Ltd., Tokyo, Japan). Subsequently, platinum was vapor-deposited using a sputtering device (JUC-5000; JEOL, Tokyo, Japan), subjected to conductive treatment, and observed under SEM (JXA-8530FA; JEOL).

### 4.8. Confirmation of the Cooling Effect on the Root Surface during Laser Irradiation

Two-thirds of the crowns of extracted human mandibular premolars were removed, the pulp chamber was opened, and the root canals were cleaned and shaped up to #60 with a K file using conventional methods. The root canals were chemically cleaned with sodium hypochlorite solution and EDTA solution and sterilized in an autoclave as described above. ICG-Nano/c solution (10 mg/mL) was injected into the root canals of the experimental group, and sterile saline was injected into the root canals of the control group. Irradiation was performed with or without air-cooling under the conditions described in Section 4.6. During laser irradiation, the temperature of the tooth root surface was measured with a thermographic camera (InfReC Thermo GEAR G100; Nippon Avionics, Yokohama, Japan) at a shooting distance of 15 cm. The minimum detection limit was 270 µm at this shooting distance. The frame time was 60 Hz and thermal images were recorded every 10 s.

### 4.9. Measurement of the Light Energy Transmitted through the Tooth Root

Tooth root samples were prepared as described in Section 4.8. A laser irradiated the samples from the top of the roots, and the transmitted light that passed through the root was measured with a power meter (Nova; Ophir Optronics Solutions Ltd., Jerusalem, Israel) set at the apex. ICG-Nano/c solution (10 mg/mL) was injected into the root canals of the experimental group, and sterile saline was injected into the root canals of the control group. The tip of the laser probe was placed above the upper edge of the root canal so that the guide circle was matched to the outer circumference of the root. The irradiation conditions were the same as described in Section 4.6. The value displayed on the power meter was recorded every 5 s.

### 4.10. Statistical Analysis

The data were tested for normality (Shapiro–Wilk and Kolmogorov–Smirnov), and a normal distribution was confirmed. Therefore, the data were further analyzed with a parametric test (Tukey’s test) to compare irradiation conditions and CFU counts, using SPSS v15.0 (IBM, Armonk, NY) with significance accepted at *p* < 0.05.

## 5. Conclusions

In this in vitro study, we present an aPDT/PACT method using ICG-loaded nanospheres coated with chitosan for the treatment of infected root canals. We produced a biofilm of the endodontic pathogen *E. faecalis* in an infected root canal model and examined the bactericidal effect on the biofilm. Our results showed that the viable cell counts of *E. faecalis* were reduced by more than 98% without an unsafe temperature rise in the root. Morphological observation by SEM confirmed a clear reduction of biofilm on the dentin block, but the removal was not complete. The laser light transmitted through the roots increased over time and had almost reached a maximum at 1 min, likely because of the consumption of photosensitizer in the root canal. For future clinical application of this method, it will be necessary to improve the bactericidal activity, perhaps by injecting the photosensitizer multiple times.

## Data Availability

The data presented in this study are available on request from the corresponding author.

## Appendix A

The tables below show the actual values of data indicated in Figure 1, Figure 2 and Figure 3.

**Table A1:** Bactericidal effect of aPDT/PACT using ICG-Nano/c on planktonic *E. faecalis*. Actual values of data indicated in Figure 1 are shown. (**a**) Bactericidal effect with irradiation of different durations. The diode laser was applied with 0.7 W peak power output without air-cooling. (**b**) Bactericidal effect with different irradiation powers. The laser operated for 5 min without air-cooling.

a	Control	1 min, 0.7 W	3 min, 0.7 W	5 min, 0.7 W
CFU/mL Average	1.19 × 10^8^	2.31 × 10^7^	7.33 × 10^5^	2.00 × 10^5^
SD	0.18 × 10^8^	0.41 × 10^7^	3.05 × 10^5^	2.00 × 10^5^
log10	8.07	7.36	5.87	5.3
log reduction		0.71	2.21	2.77
% reduction		80.59	99.38	99.83
**b**	**Control**	**0.7 W, 5 min**	**1.4 W, 5 min**	**2.1 W, 5 min**
CFU/mL Average	1.21 × 10^9^	2.31 × 10^7^	8.00 × 10^3^	1.33 × 10^3^
SD	0.26 × 10^9^	0.41 × 10^7^	7.21 × 10^3^	0.41 × 10^3^
log10	9.09	7.10	3.90	3.12
log reduction		1.99	5.18	5.96
% reduction		98.98	99.9993	99.99989

**Table A2:** Confirmation that the bactericidal effect is due to aPDT/PACT. Actual values of data indicated in Figure 2 are shown. (**a**) Comparison of bactericidal effects with and without photosensitizers. The laser was applied at 2.1 W for 5 min without air-cooling. (**b**) Bactericidal effect with air-cooling (2 L/min). The laser was applied for 5 min.

a	Control	Laser Only	ICG-Nano/C with Laser
CFU/mL Average	5.58 × 10^8^	8.29 × 10^8^	3.33 × 10^2^
SD	3.50 × 10^8^	1.65 × 10^8^	1.15 × 10^2^
log10	8.74	8.92	2.52
log reduction		−0.17	6.22
% reduction		−48.4	99.99994
**b**	**Control**	**0.7 W, 5 min**	**2.1 W, 5 min**
CFU/mL Average	8.00 × 10^7^	4.80 × 10^6^	3.27 × 10^3^
SD	1.69 × 10^7^	2.98 × 10^6^	3.12 × 10^3^
log10	7.9	6.88	3.51
log reduction		1.22	4.38
% reduction		94.0	99.9959

**Table A3:** Bactericidal effect of aPDT/PACT with IGC-Nano/c on *E. faecalis* biofilm in an infected root canal model. Actual values of data indicated in Figure 3 are shown. Lasers with different powers were applied for 5 min with air-cooling.

	Control	0.7 W, 5 min	2.1 W, 5 min
CFU/mL Average	2.63 × 10^5^	1.60 × 10^4^	3.40 × 10^3^
SD	1.46 × 10^5^	0.4 × 10^8^	2.15 × 10^3^
log10	5.42	4.2	3.53
log reduction		1.21	1.88
% reduction		93.93	98.70
